# CGull: A Non-Flapping Bioinspired Composite Morphing Drone

**DOI:** 10.3390/biomimetics9090527

**Published:** 2024-08-31

**Authors:** Peter L. Bishay, Alex Rini, Moises Brambila, Peter Niednagel, Jordan Eghdamzamiri, Hariet Yousefi, Joshua Herrera, Youssef Saad, Eric Bertuch, Caleb Black, Donovan Hanna, Ivan Rodriguez

**Affiliations:** Department of Mechanical Engineering, California State University, Northridge, CA 91330, USA

**Keywords:** sweep-morphing, morphing drones, biomimetic designs, composite materials

## Abstract

Despite the tremendous advances in aircraft design that led to successful powered flights of aircraft as heavy as the Antonov An-225 Mriya, which weighs 640 tons, or as fast as the NASA-X-43A, which reached a record of Mach 9.6, many characteristics of bird flight have yet to be utilized in aircraft designs. These characteristics enable various species of birds to fly efficiently in gusty environments and rapidly change their momentum in flight without having modern thrust vector control (TVC) systems. Vultures and seagulls, as examples of expert gliding birds, can fly for hours, covering more than 100 miles, without a single flap of their wings. Inspired by the Great Black-Backed Gull (GBBG), this paper presents “CGull”, a non-flapping unmanned aerial vehicle (UAV) with wing and tail morphing capabilities. A coupled two degree-of-freedom (DOF) morphing mechanism is used in CGull’s wings to sweep the middle wing forward and the outer feathered wing backward, replicating the GBBG’s wing deformation. A modular two DOF mechanism enables CGull to pitch and tilt its tail. A computational model was first developed in MachUpX to study the effects of wing and tail morphing on the generated forces and moments. Following the biological construction of birds’ feathers and bones, CGull’s structure is mainly constructed from carbon-fiber composite shells. The successful flight test of the proof-of-concept physical model proved the effectiveness of the proposed morphing mechanisms in controlling the UAV’s path.

## 1. Introduction

Although the first successful engine-powered heavier-than-air aircraft, the *Wright Flyer*, was fully designed and built by the Wright brothers, its design was influenced by the work of other pioneers who preceded them in their attempts to fly. Wilbur Wright wrote a short article before his death in 1912 praising Otto Lilienthal for his great contributions to aviation, considering him a hero [1]. Lilienthal conducted a comprehensive scientific study of how birds fly and flew a series of hang gliders between 1891 and 1896, becoming the first man to fly. Otto and his brother Gustav Lilienthal were deeply inspired by birds and, later, pushed the design of their hand gliders closer to bird flight by incorporating flapping wings. Otto recognized the superiority of the curved surfaces observed in bird wings and then developed a theory of flight. The first written appearance of the lift coefficient equation was in Lilienthal’s book ‘Bird Flight as the Basis of Aviation: A Contribution Towards a System of Aviation’ [1,2]. The technologies and materials that existed at the age of the aviation pioneers limited their ability to be inspired by birds in all aspects of the design. Additionally, the separation of the three key functions of lift generation, propulsion, and control directed the development of fixed-wing aircraft design for the next century, without a lot of further inspiration from nature [3]. Accordingly, today’s traditional aircraft feature rigid wings for lift generation, a jet engine or propeller for propulsion, and discrete lifting surfaces for stability and control. This contrasts with birds’ techniques of flight, which feature flexible wings that flap and morph to generate lift, propulsion, stability, and control. Although the current technologies and developments realize aircraft with capabilities that exceed those of birds, such as supersonic speeds, vertical takeoff and landing, and large payloads, there are yet more lessons to be learned from birds in their highly efficient flight without engines, propellers, or flight control surfaces [4]. Especially when designing unmanned aerial vehicles (UAV), whose missions resemble those of birds in their daily lives, searching for food and moving from place to place with various possible disturbances and obstacles, a bioinspired design approach influenced by birds’ flight can result in capabilities nonexistent in traditional drones. Yet, realizing such designs is limited by advances in actuators and lightweight materials. Hence, designs that were thought to be impossible in the past can now be achieved and will be further refined in the future with more improvements in the current technologies.

Designing a UAV with morphing wings is indeed more complex than designing a traditional UAV due to the need for an efficient actuation system for the morphing deformation, which should not significantly impact the structural integrity of the UAV. A lot of morphing wing designs were proposed to replicate one aspect of deformation observed in birds’ wings [5,6]. Examples include span-morphing [7,8], twist-morphing [9,10], and camber-morphing [11,12,13] wing designs. Most of these designs lacked important features in avian wings, such as the location of the joints, the presence of feathers, and the spanwise variation in the wing profile. Birds have different degrees of freedom in their wings, giving them advanced capabilities to change the shape of their wings. However, introducing more than one morphing degree of freedom can complicate the design and compromise the structural integrity of the wing. Only a few of the proposed wing designs were assembled into a fuselage or designed as a subsystem of a full UAV. Examples of morphing UAV designs include the Transformer aircraft [7], MataMorph-2 [10], and MataMorph-3 [13]. Not all of these morphing UAV designs featured morphing tails or had bioinspired fuselage or tail designs. Morphing wings were retrofitted to a traditional fuselage design in many reported UAV designs, with possibly a traditional tail design [7].

Birds hold a distinct advantage over fixed-wing aircraft, especially when it comes to agility and adaptability [14]. However, creating UAV designs that mimic all biological features of birds, such as bones, joints, muscles, skin, and feathers, remains an engineering challenge from the structural and control points of view. Bioinspired flapping and non-flapping UAVs have been reported mainly in the last decade [15,16,17], with the recent advances in composite and lightweight materials, 3D-printing, and mini-servomotor technology. Non-flapping designs are more energy-efficient and less complicated than flapping designs [18]. Focusing on non-flapping designs, UAVs were designed based on different species [19], as shown in Table 1. For example, the variable gull-wing aircraft [20] or the WhoopingMAV [21] featured onboard and outboard wing dihedral morphing to perform craning deflection observed in seagulls and many other bird species. The morphing UAV design by Grant et al. [22] was also inspired by the seagull but featured independent inboard and outboard wing sweep-morphing. “MataGull” was also inspired by the seagull but combined wing sweep- and dihedral-morphing along with tail tilt, pitch, and feather spread using flexible 3D-printed mechanisms [23,24]. The design of “Lishawk” [25] was inspired by the flight of the Northern Goshawk and featured five degrees of freedom, namely independent left and right wing feather folding, tail pitch, tail yaw, and tail feather folding. “LisEagle” [26] was inspired by the flight of eagles and extended the Lishawk design by introducing wing pitching deformation to further improve the turning performance. “PigeonBot” [27] obtained inspiration from the pigeon and used real biological feathers in its sweep-morphing wings. The proposed design of the bionic albatross aircraft [28] was inspired by the albatross flight with wingtip sweep and synchronous tail extension/retraction. Tail pitch, tilt, and asymmetric feather folding were proposed in [29]. Outer wing feather spread was developed and tested in [30]. The function of the tail in these UAVs is as equally important as the wing because it plays a big role in the stability and control of the UAV. For instance, wind tunnel tests performed on the tail mechanism developed by Murayama et al. [29] showed that changing the tilt angle while having a downward elevation generates lateral forces in the yaw direction. Controlling tail tilt allowed for more control over yaw without affecting efficiency.

Harvey and Inman [31] presented a literature review on methods used to quantify the aerodynamic efficiency of gliding birds and compared them to comparable UAVs. Their survey highlighted the high efficiency of gliding birds in subcritical Reynold’s number (Re) regimes, suggesting that future avian-inspired morphing UAVs can extend their operational capabilities into lower Re ranges. The recent review on avian-inspired morphing for UAV flight control by Harvey et al. [19] highlighted the effects of wing sweep-, dihedral-, twist-, and camber-morphing as well as tail incidence, spread, and rotation on the longitudinal and lateral control of UAVs. The review also surveyed coupled wing-tail morphing designs as an emerging area of study. Focusing on the flight and inertial characteristics of 22 bird species, Harvey et al. [32] showed that birds can transition between stable and unstable states via wing morphing. They found that wing morphing allows for birds to substantially change their roll and yaw inertia but has a minimal effect on the position of the center of gravity (CG). None of the bioinspired UAV designs presented so far were able to capture all the morphing degrees of freedom that birds have control over in their wings. Each design focused on a specific degree of freedom or morphing capability to show its impact on the UAV’s performance.

This paper presents a bioinspired, non-flapping, morphing UAV, named “CGull”, which resembles the Great Black Backed Gull (GBBG), *Larus marinus*, in many physical characteristics. CGull features two coupled degrees of freedom in the wing to sweep the middle wing forward and the feathered outer wing backward while retracting the wing feathers. CGull’s tail has two modular degrees of freedom to pitch and tilt the tail mechanism. The wing features a fixed 7° dihedral angle and a 3.5° angle of incidence to represent the GBBG’s wing characteristics more accurately. Carbon fiber composites were used in manufacturing various structural components in CGull, such as the fuselage, wing covers, and feathers, to increase the specific stiffness and strength and to mimic the biological construction of the alternative bird organs that are naturally made of fibers. Actuation and flight tests were performed using a proof-of-concept model to validate the proposed design. The rest of this paper is organized as follows. Section 2 presents the preliminary computational model developed in MachUpX, followed by the details of the various subsystems, such as wing, tail, fuselage, propulsion, and avionics. The section ends with a description of the manufacturing process. Section 3 presents the actuation and flight tests performed. This is followed by a discussion in Section 4. This paper is concluded with a final summary and conclusions in Section 5.

## 2. Materials and Methods

### 2.1. Mathematical Model

The adult male Great Black Backed Gull (GBBG) was chosen as the inspiration for the target planform shape, wingspan, weight, and flight speed. GBBG weighs up to 2275 g, with a wingspan of 145 to 185 cm and a body length of 61 to 78 cm [33]. Scans of seagull wings suggest that the S1223-il airfoil closely resembles their span-averaged cross-sectional wing profile [34]. This airfoil generates a high lift at low Reynolds numbers. Since the exact species of seagull that these scans were obtained from was unspecified, it was assumed that the GBBG has a similar cross-sectional profile to the S1223-il airfoil for the purposes of creating a preliminary model. Liu S20, Liu S40, and NACA 3603 airfoils were also found to be suitable representations of the common seagull’s wing profile [34,35]. However, seagull wings cannot be accurately approximated by a single airfoil shape but rather by a distribution of different airfoils along the wingspan.

A numerical model of CGull was developed using MachUpX (https://machupx.readthedocs.io/en/latest/introduction.html, accessed on 27 August 2024), an open-source Python-based software developed by the Utah State University AeroLab [36]. MachUpX employs a numerical lifting-line algorithm with modifications based on the works of Reid and Hunsaker [37] and Goates and Hunsaker [38] to model the aerodynamic characteristics of any custom aircraft. MachUpX also has a complimentary Python module for modeling airfoils, called AirfoilDatabase. Any airfoil coordinate data can be imported into the AirfoilDatabase and utilized to calculate the corresponding airfoil properties. From these calculations, either an airfoil database, a polynomial fit, or linear coefficients describing the characteristics of the airfoil can be generated. Subsequently, any of these files can then be imported into MachUpX to describe the construction of an aircraft using the airfoil data in addition to other geometric parameters. A “scene”, or the conditions the aircraft is placed in (i.e., density, kinematic viscosity, angle of attack, and velocity), should be defined. The software then computes the aerodynamic forces, moments, and coefficients of the aircraft based on the conditions described.

**Figure 1:**
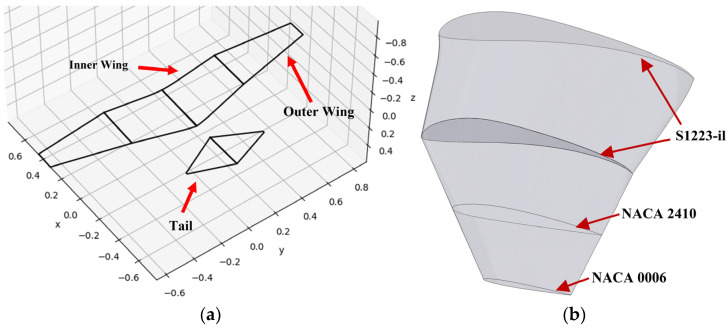
(**a**) CGull’s preliminary computational model in MachUpX; (**b**) airfoil distribution of the preliminary wing model.

Figure 1a shows the preliminary model of CGull that was defined in an aircraft object file in MachUpX as a set of wings and a tail. The reason for the exclusion of the fuselage was due to the limitations of the numerical lifting-line method MachUpX employs. This particular method is mainly intended to accurately predict the lift distribution of straight and swept wings of varying geometries [37,38]. The aircraft was defined in a three-dimensional space where the x-axis, y-axis, and z-axis correspond to the aircraft’s longitudinal, lateral (i.e., along the wingspan), and vertical directions, respectively, as shown in Figure 1a. The wings of the preliminary model were defined as two spanwise segments of inner and outer wings on the left and right sides.

The airfoil profile across the wings consisted of three linearly blended airfoils, as illustrated in Figure 1b. Starting from the root chord, the inner wing segments are assigned the S1223-il airfoil profile. The outer wings start with S1223-il and gradually transform to the NACA 2410 airfoil profile along 50% of their span, with the remainder gradually transforming into the NACA 0006 airfoil profile. The airfoils were defined by three files containing polynomial fits of each airfoil’s characteristics, which were generated using AirfoilDatabase. A fixed 7° dihedral angle was assigned to the whole wing. The NACA 2410 and NACA 0006 airfoils were selected with the eventual manufacturing process in mind. To be specific, the fully realized design of CGull was set to have artificial wing and tail feathers. The NACA 0006 was selected to represent those feathers that would be manufactured from composite materials and have a flat, uncambered geometry. The NACA 0006 is a suitable representation of these artificial feathers for its thin and symmetrical profile. NACA 2410 airfoil was also used to represent a cover surface that would be fitted over a portion of the feathers along the wingspan of the physical model. This cover was anticipated to reduce drag at the leading edge and create a smooth transition from the inner wings designed with the S1223-il airfoil in the inner wing to the outer wing made up of flat feathers.

The tail was defined as two spanwise segments mirrored on the left and right sides, and the NACA 0006 profile was assigned across its entire span. The center of gravity (C.G.) of the preliminary model was not changed from the default settings predefined by MachUpX. As such, the C.G. remained fixed to the origin of the body-fixed coordinate system, which corresponds to a quarter of the chord of the aircraft’s main wings.

**Figure 2:**
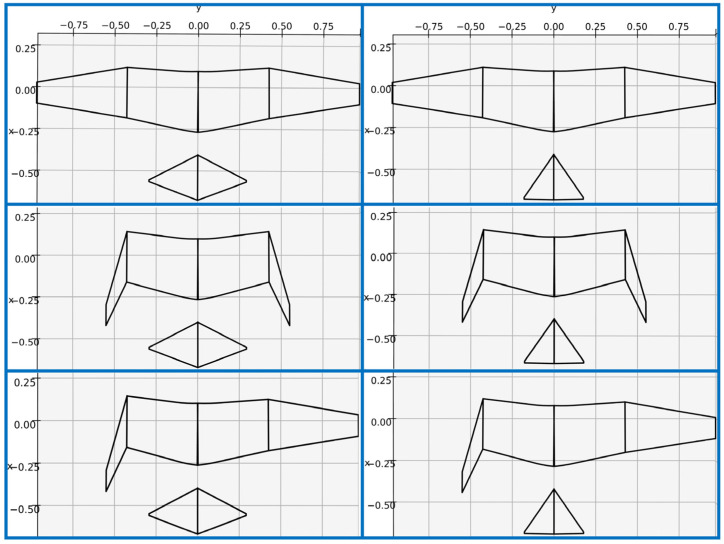
Six considered morphing wing and tail configurations.

The geometries of the wings and tail of the preliminary model were manipulated to initially produce six different configurations for a handful of wing and tail morphing combinations. These configurations, shown in Figure 2, include (a) fully extended wings with a fully extended tail, (b) fully extended wings with a tucked tail, (c) tucked wings with an extended tail, (d) tucked wings with a tucked tail, (e) asymmetric wings with an extended tail, and (f) asymmetric wings with a tucked tail. For the fully extended wing configurations, the inner and outer wings totaled a combined wingspan of 185 cm, with a planform area of 4756 cm^2^. The wingspan was reduced by approximately 57% of the extended wingspan to 106 cm for the tucked-wing configuration. The wing area decreased by approximately 34% from the extended-wing to the tucked-wing configuration, which has a planform area of 3116 cm^2^. The asymmetric-wing configurations were created by combining the left inner and outer wing segments of the tucked-wing configuration with the right inner and outer wing segments of the extended-wing configuration. As such, the wingspan and area of this configuration were 146 cm and 3936 cm^2^, respectively. The extended tail had a span of 56 cm and a planform area of 756 cm^2^, which were reduced to a span of 34 cm and an area of 459 cm^2^ for the tucked tail.

For all configurations, each wing segment was given a grid size of 90 horseshoe vortices to be modeled in the numerical lifting-line algorithm MachUpX employs. This number of vortices was found to provide a sufficient distribution of control points along the wing and tail segments as it achieved convergence of the nonlinear solver within a manageable number of iterations at the default convergence threshold. MachUpX’s nonlinear solver was employed with a convergence tolerance of 10^−10^ and a relaxation factor of 1.0 for the calculation of the aerodynamic forces and moments of each configuration.

Standard atmospheric, sea-level flight conditions (a density of 1.23 kg/m^3^ and a kinematic viscosity of 1.46 × 10^−5^ m^2^/s) were defined in the scene object file for all six wing and tail morphing configurations. The velocity of the aircraft was set to 13.4 m/s (approximately 30 mph), which is typically the highest flight speed that seagulls can reach [39]. The lift and drag forces as well as the roll, pitch, and yaw moments acting on the aircraft were recorded for an angle of attack (AOA) range of −5° to 16°. The force and moment quantities obtained were non-dimensionalized using the following equations:

(1) $$C_{L}=\frac{2L}{\rho V^{2}S};\;C_{D}=\frac{2D}{\rho V^{2}S};\;C_{l}=\frac{2M_{x}}{\rho V^{2}Sb};\;C_{m}=\frac{2M_{y}}{\rho V^{2}Sc};\;C_{n}=\frac{2M_{z}}{\rho V^{2}Sb}$$

where *C_L_* and *C_D_* are the lift and drag coefficients, respectively, and *C_l_*, *C_m_*, and *C_n_* are the roll, pitching, and yaw moment coefficients, respectively. *L* and *D* are the lift and drag forces, respectively. *M_x_*, *M_y_*, and *M_z_* are the roll, pitching, and yaw moments about the x-, y-, and z-axes, respectively. *ρ* is the air density, *V* is the aircraft velocity, *S* is the planform area of the main wings, *b* is the wingspan, and *c* is the mean wing chord.

**Figure 3:**
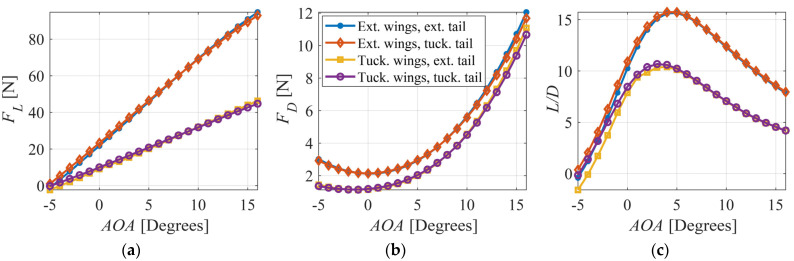
(**a**) Lift force, (**b**) drag force, and (**c**) *L/D* vs. *AOA* for four different configurations.

Figure 3a,b show the lift and drag forces, respectively, versus AOA for the four symmetric wing and tail morphing configurations, while Figure 3c shows the aerodynamic efficiency (*L/D*). It can be observed that wing extension increases both the lift and drag forces as it increases the wing area. The generated lift can balance a UAV weight of up to 22 N (2.24 kg) when the wings are extended at a 0° AOA. If the wings are tucked, a 4° AOA would be needed to balance a 2 kg weight (19.6 N). Tail extension has little effect on both forces, given the relatively smaller change in area of the tail compared to that of the wings. The maximum aerodynamic efficiency is achieved at an AOA of 5° when the wings are extended and 3° when the wings are tucked. Extending the wings can significantly change the generated aerodynamic lift and efficiency. The results are in general agreement with the findings of Ajanic et al. [25] from wind tunnel tests on LisHawk, which is a smaller bird-like UAV.

**Figure 4:**
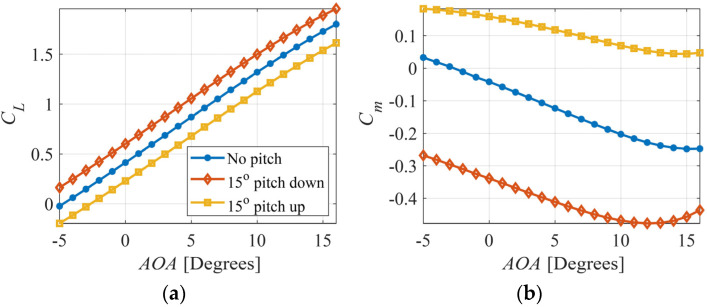
Effect of tail pitch on (**a**) lift coefficient and (**b**) pitching moment coefficient.

The effect of tail pitching is presented in Figure 4a,b. It can be observed in Figure 4a that the lift coefficient can be increased or decreased by pitching the tail down or up, respectively. This is also in agreement with the results of Ajanic et al. [25]. The pitching moment coefficient is significantly affected by tail pitch, as shown in Figure 4b. The slope of the curve is negative for all cases up to a specific AOA, indicating a stable flight. At high AOAs, the slope becomes positive, rendering the UAV unstable. This instability happens earlier when the tail is pitched down.

**Figure 5:**
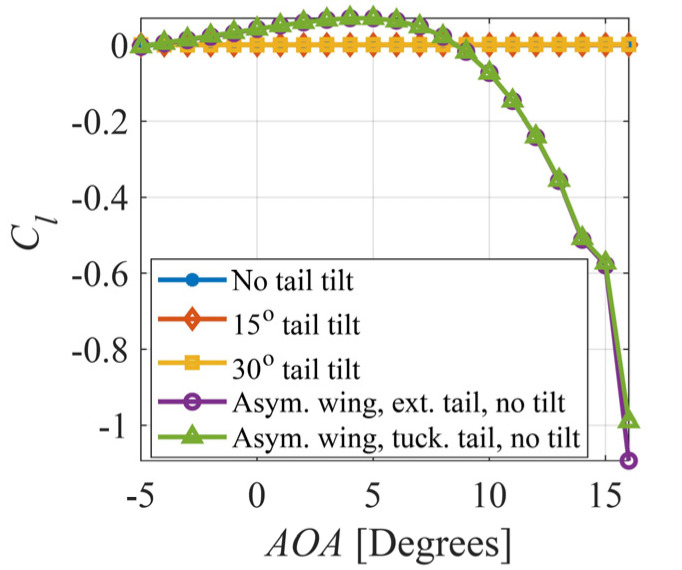
Effect of asymmetric wing morphing and tail tilt on roll moment coefficient.

The effect of asymmetric wing morphing on the roll moment coefficient is shown in Figure 5, along with the effect of tail tilt up to 30° with extended wings. Clearly, asymmetric wing morphing is much more significant on the generated roll moment compared to tail tilting.

**Figure 6:**
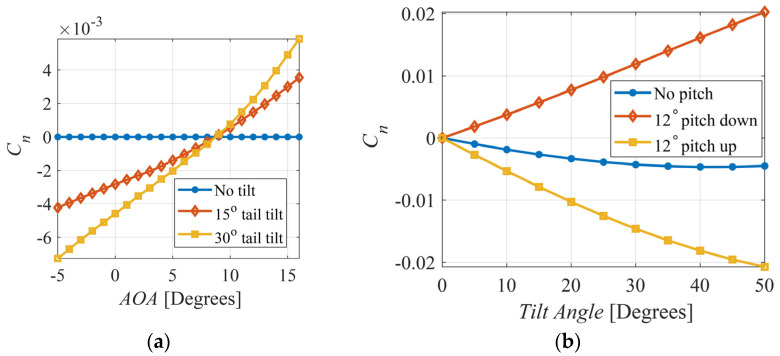
(**a**) Effect of tail tilt on yaw moment coefficient; (**b**) effect of combing tail pitch and tilt on yaw moment coefficient.

Figure 6a shows the effect of tail tilt on the generated yaw moment coefficient. Where tail tilt alone produces miniscule yaw moments, combining the tilt and pitch DOFs of the tail can lead to a much greater effect. Figure 6b shows the impact of pithing the tail down or up by 12° on the yaw moment at different tail tilt angles and 0° AOA. Having the tail pitched down in conjunction with increasing tilt angles produce a positive slope, which is desirable for stability. Also, the increasing trend of the yaw moment with rising tilt at a downward pitch angle indicates that the turning maneuverability of the UAV can also be significantly increased at greater tilt angles. This finding concurs with those of the aforementioned wind tunnel tests conducted using a bird-like tail by Murayama et al. [29].

### 2.2. Overall Design

**Figure 7:**
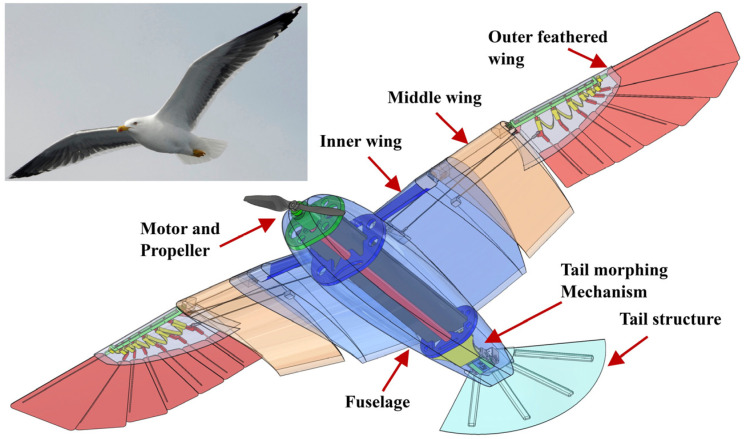
CGull’s full CAD assembly.

CGull’s full CAD assembly is shown in Figure 7. One sturdy composite structure integrates the outer shells of the fuselage and the inner wings. A linkage mechanism located inside each wing, powered by a servomotor in the fuselage, morphs each wing separately by sweeping the middle wings forward and the outer wing backward in a coupled motion. The motor and propeller assembly are secured to a front support in the fuselage. The tail structure is secured to the fuselage through a rear bracket. The tail includes two modular servomotors for pitch- and tilt-morphing. The dimensions follow those of the MachUpX model.

### 2.3. Wing Design

**Figure 8:**
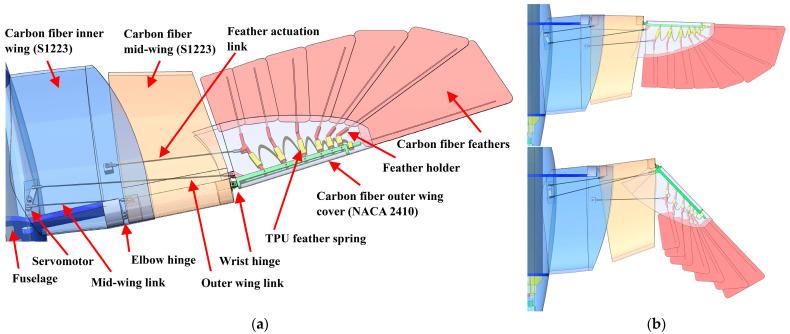
(**a**) CGull’s wing design; (**b**) wing in extended and tucked configurations.

Each of CGull’s wings is divided into three sections: a wing base or an inner wing rigidly attached to the fuselage that does not morph; a mid-wing that can sweep forward; and a distal or an outer wing that has feathers and can sweep backward, as shown in Figure 8a. The inner wing is oriented at a 7° fixed dihedral to aid in yaw stability and a 3.5° incidence to maximize lift at low speeds. The incidence angle of the wing washes out to 0° at the tip to provide stability during stall onset. The mid-wing features two hinges connecting it to the inner and outer wings. The mid-wing’s composite shell partially fits inside the inner wing’s shell for further support. A 6 kg·cm servomotor is secured in the root area of the wing, as shown in Figure 8a. This servomotor is used to actuate the two coupled morphing degrees of freedom of the wing, mimicking the movement of the elbow and wrist of the gull. Carbon fiber rods (2 mm dia.) are used as links with ball joint ends to allow for wing articulation. When the servomotor turns, it simultaneously pushes the mid-wing link and pulls the outer-wing link. This motion leads to a forward sweep of the mid-wing and a backward sweep of the outer wing. The feather actuation link connects the inner wing to the innermost feather in the outer wing. So, when morphing happens, sweeping the outer wing backwards, the feathers are retracted or tucked, decreasing the angle between the innermost feather and the outermost feather. The outermost feather stays in line with the outer-wing shell, as shown in Figure 8b, at the leading edge of the wingtip. A 3D-printed thermoplastic polyurethane (TPU) spring keeps the feathers evenly spaced while allowing for the relative motion to overlap the feathers. Wing sweep-morphing reduces the wing area and, hence, alters the generated lift and drag, enabling roll control if morphed unsymmetrically and lift and drag control if morphed symmetrically.

Carbon fiber is extensively used in the wing design of CGull due to its high specific strength and stiffness. The gull’s ten primary feathers were reduced to seven larger prepreg carbon fiber plates to reduce weight and complexity. Each feather has a carbon fiber shaft sandwiched between two carbon fiber plies. Only one of these plies takes the shape of the feather, while the other has a smaller profile and is placed on the feather shaft for reinforcement. The feather shaft does not go all the way to the feather tip to further reduce the feather weight. All wing skin sections are made of carbon fiber to act as load-carrying members between the joints. Hinge sections are 3D-printed from tough PLA and conform to the leading-edge shape of the wings to allow for large bonding areas. The outer wing’s internal structure and the feather holders are also 3D-printed from PLA.

### 2.4. Tail Design

**Figure 9:**
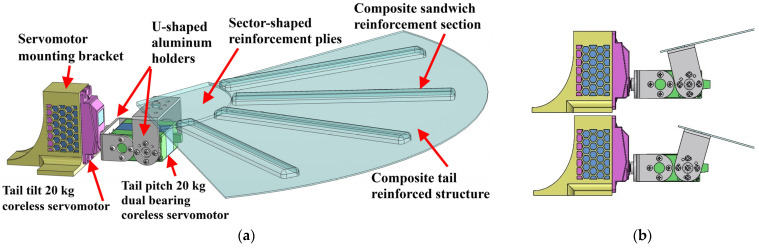
(**a**) CGull’s tail design; (**b**) tail in pitched up and down configurations.

CGull’s morphing tail implements two degrees of freedom to perform its pitch and tilt morphing motions, as shown in Figure 9a. The tail tilt 20 kg·cm servomotor is secured in a 3D-printed PLA mounting bracket. This servomotor is connected to a U-shaped aluminum holder that is secured to the 20 kg·cm tail-pitch dual bearing servomotor. Another U-shaped aluminum holder connects the tail pitch servomotor to the tail composite structure. Actuating this servomotor pitches the tail structure up and down, as shown in Figure 9b. Actuating the tail tilt servomotor rotates the tail pitch servomotor with the tail structure all together. Given that tail feather folding does not affect the generated forces and moments significantly, as was shown from the computational model’s results, the tail structure does not have individual feathers. Rather, it is just one single composite plate to avoid complexity, mitigate weight, and improve pitch stability. The plate is one carbon fiber ply reinforced with four sections of Nomex core covered by carbon fiber strips, creating a set of sandwich structures. The reinforcement sections are radially oriented. The root of the tail structure is also reinforced with three more carbon-fiber plies, having smaller sector shapes, since this part is exposed to the maximum stress under expected aerodynamic loads. In addition, this part has stress concentration around the holes of the retaining screws that secure the structure to the U-shaped aluminum holder.

### 2.5. Fuselage Design

**Figure 10:**
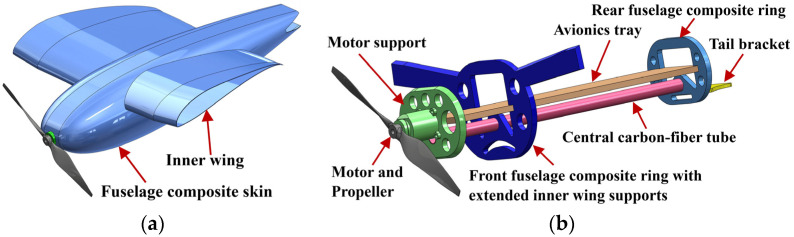
(**a**) Fuselage outer composite shell; (**b**) CGull’s fuselage internal structure.

CGull’s fuselage features a NACA0020 symmetric airfoil profile, which closely mimics the shape of the seagull’s body [35]. The profile was widened to make more room for avionics, and the inner wings were merged into the fuselage to form a single sturdy structure, as shown in Figure 10a. The internal structure of the fuselage includes front and read rings made of carbon-fiber sandwich panel with a Nomex core, connected via a central carbon fiber tube (25 mm dia.) for further reinforcement, as shown in Figure 10b. All avionic components are secured to the avionics tray, which is also supported from both sides on grooves in the front and rear fuselage rings. A motor support holding the motor is secured to the avionics tray and composite skin. The shaft of the motor runs through a hole in the nosecone of the fuselage to be connected to the propeller. The motor is slanted at 2° to the right via a 3D-printed wedge between the motor and its support structure to avoid the tendency of the UAV to roll left due to the clockwise rotation of the propeller. The tail bracket is secured to the rear fuselage ring. The top surface of the fuselage skin can be removed to access the internal components.

### 2.6. Propulsion and Avionics System

**Figure 11:**
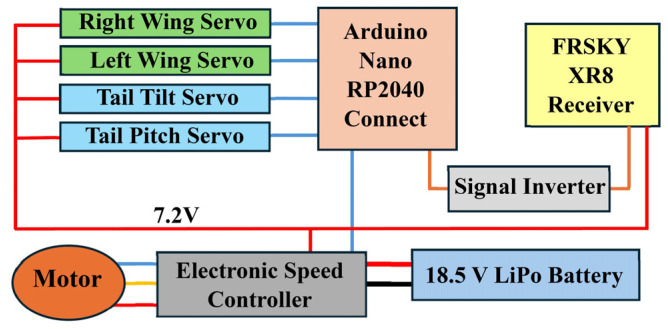
CGull’s avionics diagram.

**Figure 12:**
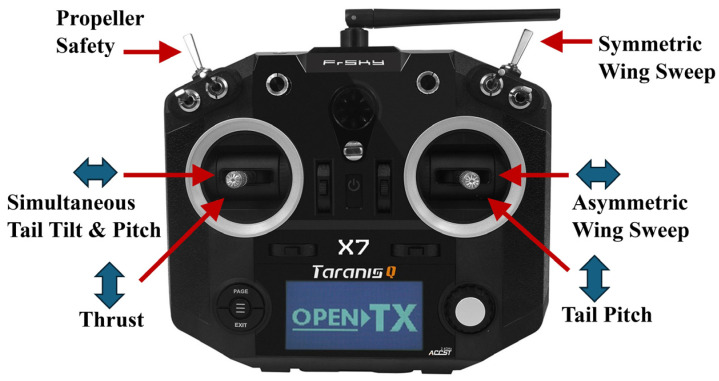
CGull’s remote control mapping.

Since CGull is a non-flapping design, a Spektrum 4240 brushless DC motor and a 12 × 6″ 2-vane nylon propeller are used to generate thrust. Electrical power is supplied by an 18.5 V LiPo battery to the Spektrum 45A Electronic Speed Controller (ESC), which controls the motor and outputs a regulated 7.2 V source to the avionics (Figure 11). The avionics system consists of a FRSKY XR8 receiver and an Arduino Nano RP2040 Connect, which runs a custom ArduinoIDE script. The XR8 receives radio signals from an FrSky Taranis Qx7 controller and sends them over SBUS to the Arduino through an inverter circuit. PWM signals are then generated by the Arduino and sent to the servo motors corresponding to the commands received. The Taranis Qx7 was mapped as pictured in Figure 12 and can be found in a typical thrust control on the left joystick as well as simultaneous tail tilt and pitch in place of the rudder control. The right joystick was mapped with tail pitch replacing the elevator and wing asymmetric sweep replacing the ailerons. A symmetric sweeping capability was also added on the right trigger, and a propeller safety shutoff was added on the left trigger.

### 2.7. Manufacturing

**Figure 13:**
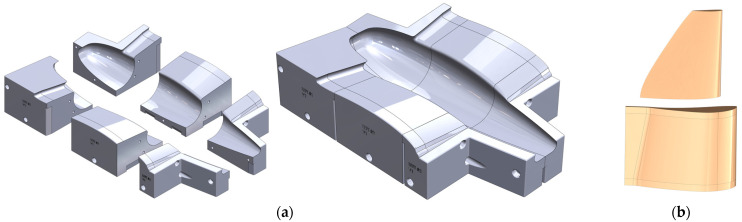
(**a**) Exploded and assembled views of the fuselage and inner-wing lower mold; (**b**) mid-wing and outer-wing skin molds.

**Figure 14:**
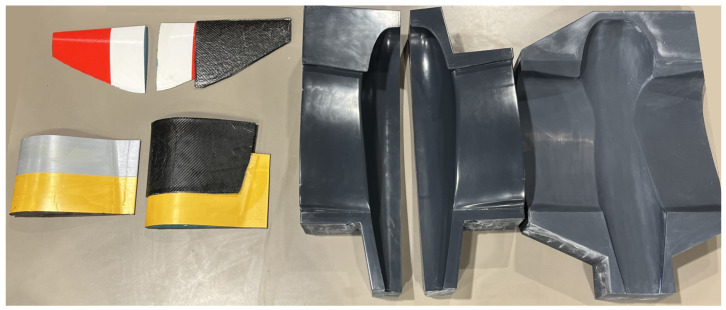
3D-printed molds used for composite manufacturing.

The wet-layup composite manufacturing technique was selected for building the fuselage and inner-wing skin shell due to the highly curved surface of this structure and the flexibility of this manufacturing technique. Upper and lower female molds were first constructed individually. Each mold was divided into multiple parts that can be 3D-printed individually and then assembled using bolts. Exploded and assembled views of the lower skin mold are shown in Figure 13a. The upper skin mold was designed similarly. This approach has two benefits. First, the parts can be disconnected at the end of the cure process to facilitate the release of the composite structure. Second, any of the parts can be replaced in case they are damaged from repeated use. A high-density foam, commonly used for composite molds, would lack these two benefits, and the negative draft angle at the nosecone would have been impossible to achieve with a traditional CNC router. To improve surface finish and ease part release, the molds were smoothed with multiple steps of automotive body filler and sanded; then, a final coat of Duratec polyester surface primer was applied. The final mold preparation consisted of wet sanding at 2500 grit and 6 coats of mold release wax. Figure 14 shows the assembled and finalized lower mold and the upper mold prior to the final sanding step. Two plies of carbon-fiber weave were used for constructing the skin in the wet-layup process.

Male molds were designed and 3D-printed for the mid- and outer-wing composite skins, as shown in Figure 13b. The mid-wing skin mold had an extended trailing edge to separate the upper and lower surfaces during manufacturing for easier mold release. The molds were sanded to obtain a smooth surface finish and then totally covered by a release film. Two plies of woven carbon fiber were used in the wet-layup process. Post-processing, after curing and mold release, all edges were trimmed, bringing each skin section to its desired size, and the upper and lower surfaces were bonded at the trailing edge. Figure 14 also shows these molds with the mid- and outer skins placed on them for demonstration.

**Figure 15:**
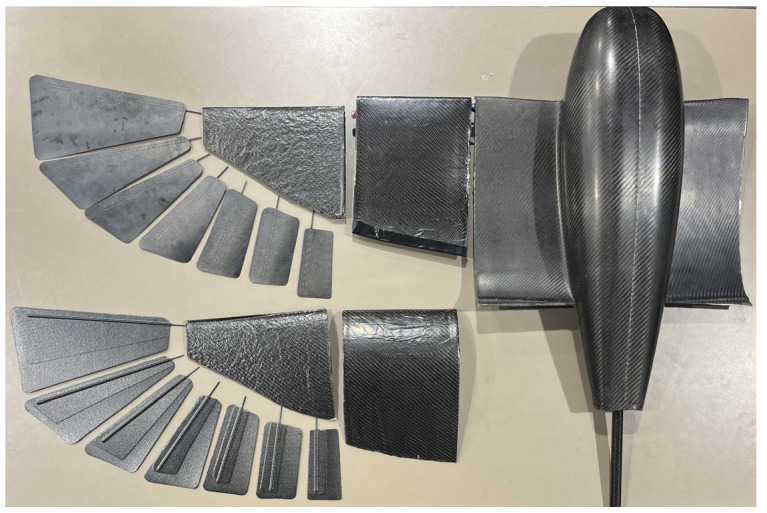
Composite structures of the fuselage and wings.

Feathers were manufactured from two plies of CYCOM 5350 woven carbon fiber prepreg sandwiching 2 mm dia. carbon fiber rods that act as feather shafts. To reduce weight, the top layer has a smaller surface area than the bottom one, and the shafts were not extended all the way to the feather tip, as shown in Figure 15. This construction reinforced the central area of the feathers around the shafts. A flat, tempered glass plate was used as a mold. The vendor-recommended cure cycle was followed in an autoclave. All manufactured composite structures for the wing and fuselage skin are demonstrated in Figure 15. The fuselage rings, the avionics tray, and the tail bracket were cut from a sandwich composite panel that has two plies in each face plate and a honeycomb Nomex core (5 mm thick). All other components were 3D-printed and assembled. The total weight of the tail subassembly is 371 g, and the fuselage with wings and avionics altogether weighs 1158 g. The total UAV weight is 1529 g, which can be carried by the generated lift of 0° AOA even in the tucked configuration.

**Figure 16:**
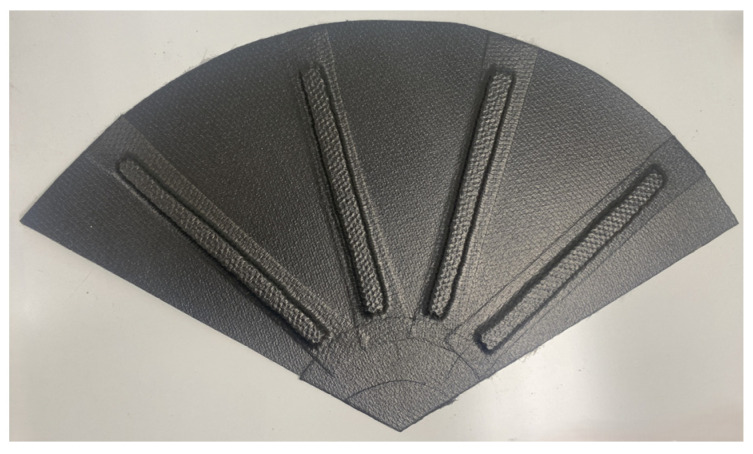
Manufacturing of the final composite tail model.

The tail reinforced structure was constructed using two plies of CYCOM 5350 woven carbon fiber prepreg reinforced with sections of 3 mm Nomex sandwich structures, as shown in Figure 16.

## 3. Results

### 3.1. Actuation Testing

**Figure 17:**
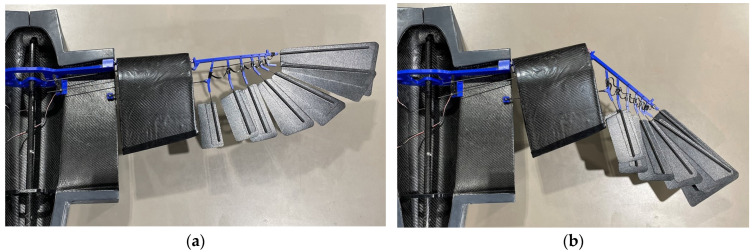
Wing actuation: (**a**) extended wing, (**b**) tucked wing (the fuselage and inner-wing lower-skin structure are placed on its mold; the top skin is removed for demonstration; and the outer-wing composite skin is also removed to show the feather folding mechanism).

A wing actuation test is shown in Figure 17a,b. Figure 17b demonstrates the mid-wing moving forward, while the outer wing moves aft upon actuation of the servomotor in the fuselage. The coupled wing-sweep mechanism was able to effectively produce the intended area change in the wings with the selected servomotors.

The tail pitch and tail tilt servomotors were also able to produce the desired tail morphing actuation. The tail pitch servomotor was able to change the tail elevation angle to approximately 15° upward and 15° downward from a fixed horizontal position. The tail tilt servomotor was able to alter the tilt angle of the tail up to 30° in either clockwise or counterclockwise directions. Another actuation test was conducted in which CGull was placed on a smooth and flat surface that would allow for it to rotate but not translate. The wings were extended, and the motor with its attached propeller was subsequently turned on. The tail was then pitched downward and tilted. When the tail was tilted in a clockwise direction, the UAV pivoted on the flat surface in such a way that the extended right wing moved forward, turning the UAV left as viewed from its perspective. The opposite effect was produced by tilting the tail counterclockwise. This test manifested the effect of the simultaneous tail pitch and tail tilt on the generated yaw moment that had been observed from the computational model.

### 3.2. Flight Test

**Figure 18:**
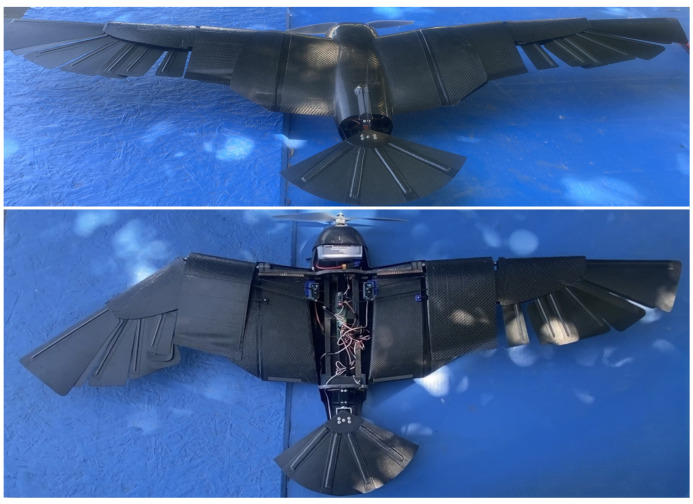
CGull prototype before the flight test (the top fuselage cover is removed, and the left wing is swept back in the bottom figure).

The manufactured CGull’s prototype is shown in Figure 18. All movable components stored in the fuselage on the avionics tray were moved as far forward as possible. This adjustment allowed for the CG of the drone to be closer to the quarter of the mean aerodynamic chord of the wings. Videos S1 and S2 in the Supplementary Materials show the actuation tests conducted before the flight test.

**Figure 19:**
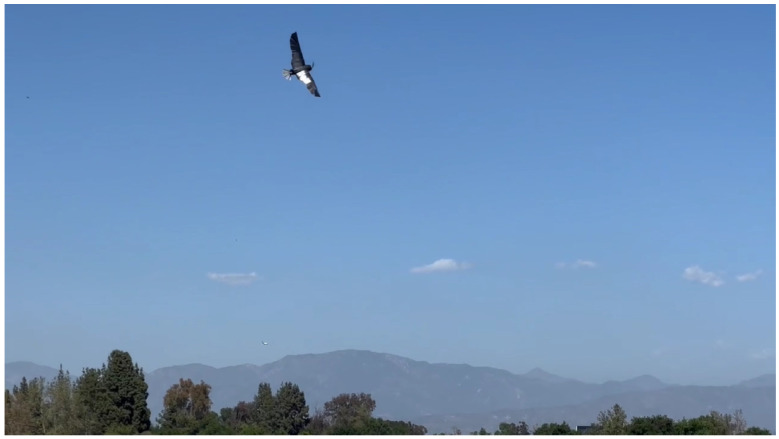
CGull’s flight test.

CGull’s prototype was taken to Apollo VI Airfield in Lake Balboa, CA, to be flight-tested by a certified drone/RC plane pilot. Figure 18 shows the drone before the flight test. To minimize any potential damage during landing, the landing process was planned to be executed by slowing down the UAV and allowing it to slide on its belly on mowed grass. After the pilot became familiar with CGull’s flight controls, the drone was launched via a hand toss into the air. CGull was able to perform a successful flight, reaching a considerable altitude from its starting position near the ground, as shown in Figure 19, and completing a full loop back. Eventually, CGull started to encounter instability due to the vibration of the carbon fiber wing feathers. The feathers themselves are very stiff, but their 3D-printed PLA connectors were not stiff enough to keep them rigid under the applied aerodynamic loads. Despite this, the pilot stated that controlling CGull was quite manageable, especially for a nonconventional aircraft that does not resemble any UAV flown in this airfield before. Video S3 in the Supplementary Materials shows the flight test.

## 4. Discussion

RC pilots are used to control UAVs with ailerons, rudders, and elevators for roll, yaw, and pitch control, respectively. For the proposed nontraditional UAV, CGull, the only control degree of freedom that matches a traditional flight control surface is tail pitch for pitch control. The other two degrees of freedom, namely asymmetric wing sweep for roll control and simultaneous tail tilt and pitch for yaw control, are nontraditional. Incorporating nontraditional UAV designs in flight simulators can help RC pilots practice flying such new designs, since practicing in physical flight tests directly may not give enough time for testing different control actions. The performance of LisHawk [25], which is a significantly smaller bioinspired drone, was studied using wind tunnel tests where the whole drone was placed inside a wind tunnel whose test section was large enough to fit the whole model, computational studies, and flight tests. With LisEagle [26], which is a bioinspired drone whose size is between LisHawk and CGull, an open-jet wind tunnel was used since a crash could lead to a setback of weeks or even months, with the risk of major repairs compromising repeatability [40]. Given the larger size of CGull and the lack of an open-jet wind tunnel facility, before the flight test, CGull’s performance was only assessed using the computational model and the actuation tests. The success of the first flight test validated the proposed design and design choices.

Future iterations would consider reinforcing the feather connectors to avoid the encountered feather flutter problem and performing more flight tests. Given that the wing includes an actuator and moving parts, such as feathers and linkages, that experienced vibration under aerodynamic loads during the flight test, an aeroelastic analysis of this wing can also be conducted to guide the design process. Optimization studies can also be conducted to identify the optimal values of the design variables. Further tests should also be conducted to evaluate the reliability and operational life of each morphing mechanism under the expected applied loads and validate the decision of the actuators. An onboard monitoring system can also be integrated into the drone to collect data reflecting the actual performance and compare it with the nominal performance. Optimization studies can be performed for further weight reduction.

## 5. Summary and Conclusions

This paper presents a bioinspired non-flapping UAV, named “CGull”, that features a significant number of composite structures. A numerical model created in MachUpX was first developed to understand the effects of the various morphing degrees of freedom on the generated aerodynamic forces and moments. The various subsystems were described, including wing design, tail design, fuselage design, propulsion, and avionics systems. The manufacturing process was also described. The physical proof-of-concept model of CGull was built and successfully passed the actuation test and the first flight test. The proposed morphing mechanisms proved to be effective in generating the required forces and moments to fully control the UAV. The chosen actuators and avionics components also proved to be adequate for this application. CGull is the first composite seagull-inspired drone with feathered wings that have two coupled sweep-morphing degrees of freedom along with a rudderless tail.

## Figures and Tables

**Table 1:** Wing and tail morphing capabilities in bioinspired non-flapping UAV designs.

Design	Bird	Wing Morphing	Tail Morphing
Variable gull-wing aircraft [20] or “WhoopingMAV” [21]	Seagull	Wing craning deflection (inboard and outboard dihedral morphing)	Traditional
Grant et al. [22]	Seagull	Independent inboard and outboard wing sweep	Traditional
“MataGull” [23,24]	Seagull	Feather folding and dihedral morphing	Pitch, tilt, and feather folding
“Lishawk” [25]	Northern Goshawk	Feather folding	Pitch, yaw, and feather folding
“LisEagle” [26]	Eagle	Wing pitch and feather folding	Pitch, yaw, and feather folding
“PigeonBot” [27]	Pigeon	Feather folding	Traditional elevator and rudder
Bionic albatross aircraft [28]	Albatross	Wingtip sweep	Synchronous extension and retraction

## Data Availability

The data presented in this study are available on request from the corresponding author.

## Supplementary Materials

The following supporting information can be downloaded at: https://www.mdpi.com/article/10.3390/biomimetics9090527/s1, Video S1: Pre-flight actuation test; top view, Video S2: Pre-flight actuation test, Video S3: CGull’s flight test.
